# Women’s knowledge and attitude towards cervical cancer preventive measures and associated factors In South Gondar Zone, Amhara Region, North Central Ethiopia: a cross-sectional study

**DOI:** 10.1186/s13690-021-00659-4

**Published:** 2021-07-23

**Authors:** Yilkal Tafere, Tezera Jemere, Tsion Desalegn, Addisu Melak

**Affiliations:** 1 Department of Public Health, College of Health Sciences, Debre Markos University, Debre Markos, Ethiopia; 2 Department of Pharmacology School of Pharmacy, College of Medicine and Health Sciences, University of Gondar, Gondar, Ethiopia; 3 Department of Public Health, College of Health Sciences, Debre Tabor University, Debre Tabor, Ethiopia; 4 Department of Biomedical Sciences, College of Health Sciences, Debre Tabor University , Debre Tabor, Ethiopia

**Keywords:** Cervical cancer, Knowledge, Attitude

## Abstract

**Background:**

Cervical cancer is a leading cause of morbidity and mortality among women in Ethiopia, often due to late disease diagnosis. Early prevention of cancer has been shown to be the most effective measure against the disease. Scientific evidences indicate that lack of awareness towards cervical cancer is a barrier to prevention strategies. Therefore, the aim of the current research was to assess women’s knowledge and attitudes towards cervical cancer preventions in South Gondar zone.

**Methods:**

A community-based cross-sectional study was carried out in South Gondar zone, Ethiopia. The study sample comprised 844 women ≥ 18 years of age. Participants were selected using systematic sampling technique. Binary and multivariable logistic models were used to assess predictors of women’s knowledge and attitude towards cervical cancer.

**Results:**

About 66 % of the women had heard about cervical cancer. Regarding the main source of information of respondents, 75.4 % were heard from health professionals. Sixty two point 4 % of women knew at least one preventive measure and 82.6 % of participants knew at least one symptom or sign. Among study participants, 25 and 64 % had good knowledge, and favorable attitude towards cervical cancer prevention measures, respectively. Being reside in rural (AOR = 0.21, 95 %CI; 0.18, 0.34), not attending formal education (AOR = 0.50, 95 % CI: 0.3, 0.75), low income (AOR = 0.57, 95 % CI: 0.43, 0.81) and having < 4 children ((AOR = 0.8, 95 % CI: 0.60–0.86) were negatively associated with knowledge toward cervical cancer prevention measures.

**Conclusions:**

This study found the majority of the respondents had poor knowledge about cervical cancer prevention measures. The majority of the study participants had favorable attitudes regarding cervical cancer prevention. Living in rural areas, not attending formal education low income and having less than four children was negatively associated with respondents’ knowledge towards cervical cancer prevention measures. There is needed to scale up cervical cancer prevention measures and services .Further studies are needed using strong study design.

## Plain English summary

In Ethiopia, Cervical cancer is the most common cause of hospitalization and death among women, often due to late disease stage at diagnosis. Previous evidence has shown that a lack of awareness and unfavorable attitudes towards cervical cancer are considered as barriers to the prevention of cervical cancer. A community-based cross-sectional study was carried out in South Gondar zone, Ethiopia. A total of 844 women responded to the questionnaire which makes a response rate of 100 %. About 804 (95.7 %) of the respondents were orthodox in religion. The three hundred sixty seven (43.5 %) women were housewives. Nearly half (49.9 %) of the women had a monthly income between 1001 and 2000 birr.

The percentage of women who heard about cervical cancer was 66 %. Regarding the main source of information of respondents, 75.4 % were heard from health professionals. Sixty two point 4 % of women knew at least one preventive measure and 82.6 % of participants knew at least one symptom or sign. Among study participants, 25 and 64 % had good knowledge, and favorable attitude towards cervical cancer prevention measures, respectively.

In conclusion; this study found the majority of the respondents had poor knowledge about cervical cancer prevention measures. The majority of the study participants had favorable attitudes regarding cervical cancer prevention. Living in rural areas, not attending any formal education low income and having less than four children was negatively associated with respondents’ knowledge towards cervical cancer prevention measures. There is needed to scale up cervical cancer prevention measures and services so that more women can access them irrespective of where they reside.

## Background

Cervical cancer is a type of cancer that occurs in the cells of the cervix; it affects many women worldwide and is caused by Human papillomavirus (HPV) [1, 2]. The disease can be prevented via, immunization, early detection and prompt treatment of detecting precancerous lesions [3].

From the total cases of cervical cancer globally 8 % of women are living in low-income countries and it is number one among the reproductive cancers worldwide [4, 5]. Currently, the new prevention approach is focused on immunization against HPV infection as part of primary prevention, or screening for evidence of pre-invasive lesions of the cervix, as secondary prevention [1]. Having early sexual intercourse leads a woman to be infected with HPV. Patients of cervical cancer may develop symptoms years after being infected [6].

About 28 % of Cameroonian women had prior knowledge of cervical cancer [7]. A study done in Nigeria revealed that 15 % had heard of cervical cancer, overall 40 % of the respondents had poor knowledge of cervical cancer [8]. A study conducted in Uganda revealed that 55.4 % of the women had high knowledge about cervical cancer. With regards to a woman’s risk of developing cervical cancer: about 78.4 % of respondents stated that a woman might have a risk of developing cervical cancer if women had multiple sexual partners, 88.4 % being infected with the HPV, and 73.9 % respondents thought early sexual intercourse at a young. Nearly 79.2 % of women also assumed that using contraceptives for a long time increased one’s risk of developing cancer [9].

Urban resident and higher income were associated with the women’s level of knowledge about cervical cancer prevention [9]. Study in Kenya suggests that 51 % of the respondents were aware of cervical cancer; Health professionals were used as the major source of awareness for women [10]. A study in Mizan Tepi revealed that 33.97 % of the respondents had poor knowledge and only 25.4 % of the respondents had good knowledge. More than half (61 %) of women had a positive attitude for the early intervention of cervical cancer via screening [11].

In Ethiopia; Patients came for treatment at the late stage of cervical cancer in that no benefit can be obtained from any treatments. Currently, it becomes a major problem for women, as more of them keep on presenting with cervical cancer at a later stage [12]. Knowledge and attitude are important in improving the understanding of factors influencing the involvement of women in preventive activities towards cervical cancer. As far as the researcher’s knowledge, there are limited studies in Ethiopia and no studies in the study area. Therefore the objective of the study was to assess knowledge, attitude towards cervical cancer prevention measures of women in South Gondar zone, Ethiopia.

### Methods

### The Study Setting and population

This community-based cross-sectional study was conducted in South Gondar zone, Ethiopia from August 20 to September 20 /2019 in South Gondar zone. South Gondar zone is located 105 km far from Bahir Dar (the capital city of the Amhara region) and about 666 km northwest from Addis Ababa the capital of Ethiopia. According to the 2007 census projected result, south Gondar zone has populations of 2,526,813 of this 1,260,880 male and 1,265,933 were female [13].

The sample size was determined by using a single population proportion. Considering that the proportion of knowledge towards cervical cancer prevention is 50 %. For determining the sample size of this study, 5 % level of significance (a = 0.05), 5 % margin of error (d = 0.05) and 10 % non-responsive rate and design effect of 2 were taken into account. Based on this, the total sample of the study was determined to be 844.

Multi-stage sampling was used, out of twelve districts in South Gondar zone three districts was selected by lottery method. Out of the three districts, 15 kebeles (the smallest administrative unit in Ethiopia) were selected by using simple random sampling. House to house census was conducted to know the total population of women aged 18 and above in the selected kebeles. Based on the census result, 844 women were allocated proportionally to each selected kebeles Finally, individual women age 18 and above were selected using a systematic sampling technique (dividing the total eligible women in the selected kebeles to the sample size, then we took every k^th^). women were contacted directly door to door in the community. If the woman was not at home during the visit, two additional attempts were done before declaring not available .Face to face interview was administered by a nurse after explaining the objectives when women were free and in a comfortable condition at their home. Data were collected using structured and pre-tested questioners. The questionnaires were first prepared in English, and translated into the local language (Amharic).

The dependent variable was - Knowledge (good vs. Poor), -Attitude (positive vs. negative). The independent variables were age, marital status, education, religion, ethnicity, occupation Condom use with first sex, no of a partner, number of children.

Operational definitions: Good Knowledge: respondents who answer four and correct responses of knowledge questions, Poor knowledge, respondents with answered less than four correct responses of knowledge questions. Positive attitude: Respondents who scored above the mean and the mean score had a Positive attitude towards prevention for cervical cancer.

Negative attitude: Respondents who scored below the mean score had a negative attitude towards prevention for premalignant cervical cancer.

The data was entered using EP info 7 and analyzed using IBM SPSS statistics version 20 software (Armonk NY, IBM corp, USA, 2019). Logistic analysis was applied, Odds ratios with 95 % confidence intervals were computed to identify the presence of association and statistical significance is p < 0.05.

 Ethical clearance was obtained from research evaluation and ethical review committee of Debre Tabor University College of Health Sciences. Permission letter was also obtained from South Gondar Health department. The participants were informed that participation is made entirely voluntarily and verbal consent was obtained.

## Results

A total of 844 women responded to the questionnaire which makes a response rate of 100 %. The mean age of the respondents was 30.2 years (Standard Deviation [SD] = 7.3). About 804 (95.7 %) of the respondents were orthodox in religion and all (100 %) of the respondents were Amhara in ethnicity. About 618 (73.2 %) of the women were married. Regarding their occupation 367 (43.5 %) were housewives. With regard to their educational status 499(59.1 %) of women had attended formal education and the remaining 345 (40.9 %) did not attend formal education. About 60.1 % (514) of the respondent lives in rural areas. Nearly half (49.9 %) of the women had a monthly income between 1001 and 2000 birr (Table 1).

**Table 1:** The socio-demographic characteristics of women in selected districts of south Gondar zone Amhara region, north-central Ethiopia, 2019(*N* = 844)

Variables	Frequency	
**Religion**	No	(%)
Orthodox	808	95.7
Muslim	36	4.3
**Educational status**		
Not attend formal education	345	40.9
Primary	499	59.1
**Occupation of the respondent**		
Housewife	367	43.5
Farmer	166	19.7
Government employee	160	19
Other^a^	151	17.9
**Marital status**		
Single	146	17.3
Married	618	73.2
Divorced	57	6.8
Widowed	23	2.7
**Resident**		
Rural	514	60.1
Urban	330	30.9
**Family average monthly Income**		
<=1000	150	19.8
1001–2000	378	49.9
> 2000	230	30.3

^a^Other includes student, daily laborer merchant

### Knowledge about cervical cancer prevention measures

About two-thirds (66 %) of the study participants replied that they were aware of cervical cancer. Nearly three forth of the women has got the cervical cancer prevention information from the health professionals .About 59 (10.6 %) of respondents’ were used mass media as a source of information. Regarding the prevention and treatment of cervical cancer 120(14.2 %) of respondents reported that primary prevention is crucial, nearly one in ten women were aware of the curability of the disease if it is detected early. Among the respondents, 250 (29.6 %) correctly answered a minimum of one prevention measure of the disease.

One hundred fifty (17.8 %) respondents knew a minimum of one symptom of cancer. Six (0.7 %) women responded that having more than one sexual partner, infected with the human papillomavirus (HPV) 8 (1 %), practice early sexual intercourse 54 (6.4 %), and cigarette smoking 170 (20 %) increased risk of developing cervical cancer. One in hundreds of women were not aware of the risk factors for cervical cancer. Vaccination 25 (3 %), delaying sexual intercourse 68 (8 %), minimize multiple sexual relationships 40 (4.7 %), using condom 28 (3.3 %) were mentioned by the women to prevent cervical cancer. Seventy (4 %) of the respondents did not mention any prevention methods. Concerning knowledge of the respondent about signs and symptoms of cervical cancer, vaginal bleeding 110(13.1 %) and vaginal discharge 40 (4.8 %) were known by the women. Overall 212(25.12 %) of the women had good knowledge about cervical cancer prevention measures (Table 2).

**Table 2:** Knowledge women about cervical cancer and its preventive measures, in Selected districts of South Gondar zone, Amhara region North central Ethiopia,2019 ( N = 844)

	Variables	No	%
1	**Have you ever heard about cervical cancer?**		
	Yes	557	66
	No	287	40
2.	**Cervical cancer is curable if detected early**		
	Yes	89	10.5
	No	755	89.5
3	**Early detection of cervical cancer is helpful in its treatment**		
	Yes	120	14.2
	No	724	85.8
4	**Knew at least one risk factor for cervical cancer**		
	Yes	238	28.2
	No	606	71.8
5	**Someone can be vaccinated against cervical cancer**		
	Yes	240	24.44
	No	604	75.56
6	**Knew at least one preventive measure for cervical cancer**		
	Yes	250	29.6
	No	594	70.4
7	**Knew at least one symptom of cervical cancer**		
	Yes	150	17.8
	No	694	82.2
8	**Over all Knowledge about cervical cancer prevention measures**		
	Yes	212	25.12
	No	632	74.88

### The attitude of respondents towards cervical cancer prevention measures

 Almost half 430(50.1 %) of the respondents believed that they were at risk of developing cervical cancer and nearly half 441(48.1 %) of the respondents agree that Carcinoma of the cervix is highly prevalent in our country and is a leading cause of deaths amongst all malignancies in Ethiopia. More than half 522 (61.8 %) of the participants agree that cervical carcinoma cannot be transmitted from person to person while 38.2 % of them agreed it can be transmitted from person to person.

About 607(71.0 %) of the respondents agreed that screening helps to prevent cervical cancer. More than half 544 (64.5 %) of respondents perceived that screening causes no harm to the patient, whereas, 35.5 % agrees as it is harmful. In general 539(63.8 %) of the respondents had a favorable attitude towards cervical cancer prevention measures. About 472(59.9 %) of participants thought that screening for premalignant cervical lesions is expensive and 765(90.6 %) of the women were agreed that if screening is free and causes no harm to be screened (Table 3).

**Table 3:** Attitudes of women about cervical cancer and its preventive measures, in Selected districts of South Gondar zone, Amhara region North central Ethiopia,2019 ( N = 844)

No.	Variables	Disagree		Agree	
		**No**	**%**	**No**	**%**
1	Carcinoma of the cervix is highly prevalent in our country and is a leading cause of deaths amongst all malignancies in Ethiopia	447	51.9	441	48.1
2	Any adult woman including you can acquire cervical cancer	403	49.9	431	50.1
3	Carcinoma of the cervix cannot be transmitted from one person to another	322	38.2	522	61.8
4	Screening helps in prevention of carcinoma of the cervix	237	28.1	607	71.9
5	Screening causes no harm to the client	300	35.5	544	64.5
6	Screening for premalignant cervical lesions is not expensive	372	40.1	472	59.9
7	If screening is free and causes no harm, will you screen	79	9.4	765	90.6

#### Predictors of knowledge of women about cervical cancer prevention measures

Different variables were tested for their association with the knowledge of the women towards cervical cancer prevention measures. Place of Resident, Monthly income, Level of education and Parity of the women were associated with the level of knowledge on cervical cancer in both bivariate and multivariate analysis.

Women who live in rural areas were 79 % less likely to be knowledgeable cervical cancer prevention measures than their urban counterparts (AOR = 0.21, 95 %CI; 0.18, .34). Respondents who were not attending formal education were 50 % less likely to be knowledgeable about cervical cancer prevention measures than their counterpart (AOR = 0.50, 95 % CI: (0.3, 0.75)

Women having low monthly income (AOR = 0.57, 95 % CI: 0.43, 0.81), women having < 4 children ((AOR = 0.8, 95 % CI: 0.60 –0.86) were negatively associated with knowledge toward cervical cancer prevention measures compared to their counterpart .An odds ratio for religion and ethnicity of women was not computed as nearly all women belong to Orthodox Christian and all the women are Amhara in their ethnicity (Table 4).

**Table. 4:** Predictors of knowledge of women towards cervical cancer prevention measures in selected districts of South Gondar zone, Amhara region; north central Ethiopia; 2019

Variables	Knowledge score		COR(95 %CI)	AOR(95 %CI)
	**Good**	**Poor**		
**Place of Resident**				
Rural	80	506	1	1
Urban	132	198	0.24(0.21,0.5.3)	0.21(0.18,0.34) **
**Level of education**				
Not attend formal education	67	287	0.56(0.2,0.8)	0.5(0.3,0.75)**
Attend formal education	145	354	1	1
**Monthly household income**				
<=2000	152	406	0.6(0.4,0.82)	0.57(0.43,0.81)**
> 2000	90	140	1	1
**Parity**				
0–4	172	528	0.87(0.5,0.93)	0.8(0.6,0.86)**
> 4	40	107	1	1

** Significantly associated *P* < 0.05

## Discussion

The current study demonstrated that 60 % of the participants had heard about cervical cancer. Previous literature has shown that awareness of cervical cancer was found to be 28 % in Cameroon [7], 15 % in Nigeria [8], 51 % in Kenya [10], and 93 % in Ghana [14]. The variation might be due to differences in study populations and currently the government of Ethiopia gives emphasis for maternal health particularly for cervical cancer due to this reason the awareness of the respondent in the current study is relatively higher than the previous study. .A study in Ghana uses study groups were medical students, non-medical undergraduate students, nurses, and senior university staff might have a better understanding of cervical cancer, whereas the current study includes both rural and urban residents, all of them were not health professionals. Subsequently designing appropriate awareness creation mechanisms is crucial to address all women about cervical cancer prevention measures.

In this study, health professionals were used as the main source of information followed by radio or TV. This implies that health professionals have a key role in educating women.

In this study, 10 % of the women were knowledgeable about the capability of being cured of cervical cancer if it is diagnosed early. This knowledge gap might inhibit women from implementing cervical cancer prevention measures. Hence health promotion, education, and communication about cervical cancer prevention measures are important.

This study, the finding indicates that nearly a third quartile or (75 %) of the respondents had poor knowledge of cervical cancer prevention measures. In contrast to this finding; recent studies showed that in Mizan Tepi University, Ethiopia nearly 34 % [11] and Uganda, 44.6 % [9] of the respondents had poor knowledge about cervical cancer. University students might have access to different mass-medias that may increase their awareness level towards cervical cancer prevention measures. The current study was conducted at a community level, including the rural areas. Hence the awareness of these women about cervical cancer is minimal; there is a need to educate women on the early warning signs of cervical cancer as failure to recognize the early symptoms and signs contribute to the late presentation common in Ethiopia.

This implies that, in the absence of proper health education strategy in the health system and at a community level, hence tailored health education strategy should be implemented that can help to improve the knowledge of the respondents towards the symptoms of cervical cancer to take measures early.

Concerning the prevention of cervical cancer participants knew that cervical cancer is prevented by avoiding multiple sexual partners (4.7 %), avoiding early sexual intercourse (8 %), and quitting smoking (10.5 %), vaccination (3 %), using a condom (3.3 %) while in a study done in Sweden 62 % respondents reported that cervical cancer can be prevented by early screening and HPV vaccination [15]. This disparity could be related to an early screening and availability of the HPV vaccine and services might be provided in all health care systems in developed nations like Sweden to the population at every facility.

The current study indicates that overall 63.8 % of the respondents had a positive attitude towards cervical cancer prevention measures; a similar magnitude of attitude (61.24 %) is reported from recent studies in Mizan Taipei University, Ethiopia [11]. In this study, about 32 % of participants agreed to be screened for cervical cancer if the cost is free and has no harm health check-up, hence avoiding barriers will help in increasing the number of women participants for screening. Awareness creation activities regarding cervical cancer at a grass root level are crucial.

In this study, women residing in rural areas, not attending formal education, low-income, parity less than 4 were negatively associated with good knowledge of women for cervical cancer. This is possible because women who were living in urban may have access for health workers that can give health education for their clients; in addition to this; women who attend any formal education may have the ability to read different leaflet and other printed materials that are a key source of information about cervical cancer and its prevention measures. Women that have more than four children might have proximity to the health facilities that provide antenatal care may provide counseling services toward cervical cancer and its prevention measure. Integrating all health services with cervical cancer services would enhance awareness among women. Hence awareness creation activities should be accessible for women who reside in rural areas that enhance the knowledge of the women about the disease and prevention measures.

Women who belonged to the lower socioeconomic category were less knowledgeable about cervical cancer prevention compared to those from the high status might have different communication, mass media and this could reflect that the awareness might be increased that could increase the demand to use service-related cervical cancer. Lack of standardized questionnaire to measure the knowledge and attitude of the respondents, this might limit the comparability of the findings in different places.

## Conclusions

This study found the majority of the respondents had poor knowledge about cervical cancer prevention measures. The majority of the study participants had favorable attitudes regarding cervical cancer prevention. Living in rural areas, not attending any formal education low income and having less than four children was negatively associated with respondents’ knowledge towards cervical cancer prevention measures. Awareness creation campaigns should be held to provide comprehensive information about cervical cancer prevention and control mechanisms to women in all areas.

## Data Availability

The datasets are available from the corresponding author.

## Abbreviations

HPV: Human Papilloma Virus
KAP: Knowledge, Attitude and Practice
SD: Standard deviation
VIA: Visual Inspection of the Cervix with Acetic Acid
SPSS: Statistical Package for Social Science
WHO: World Health Organization
